# The concordance between preoperative aspiration and intraoperative synovial fluid culture results: intraoperative synovial fluid re-cultures are necessary whether the preoperative aspiration culture is positive or not

**DOI:** 10.1186/s12879-021-06721-4

**Published:** 2021-09-29

**Authors:** Hao Li, Chi Xu, LiBo Hao, Wei Chai, Fu Jun, Jiying Chen

**Affiliations:** 1 Medical School of Chinese PLA, Beijing, People’s Republic of China; 2 Department of Orthopedic Surgery, The First Medical Center, Chinese PLA General Hospital, 28 Fuxing road, Beijing, People’s Republic of China; 3 Senior Department of Orthopedics, The Fourth Medical Center of PLA General Hospital, Beijing, People’s Republic of China

**Keywords:** Periprosthetic Joint Infection (PJI), Synovial fluid culture, Joint aspiration, Total joint arthroplasty (TJA)

## Abstract

**Aims:**

Preoperative aspiration culture and intraoperative cultures play pivotal roles in periprosthetic joint infection (PJI) diagnosis and pathogen identification. But the discordance between preoperative aspiration culture and intraoperative synovial fluid culture remains unknown. We aim to determine (1) the discordance between preoperative and intraoperative synovial fluid (SF) culture and. (2) compared to intraoperative synovial fluid cultures, the sensitivity of preoperative aspiration fluid culture. Then the following question is tried to be answered: Are intraoperative synovial fluid re-cultures necessary if the preoperative aspiration culture is positive?

**Materials and methods:**

Between 2015 and 2019, 187 PJI patients managed with surgeries were included in this study. Compared to intraoperative synovial fluid culture, the sensitivity, specificity, positive predictive value (PPV), and negative predictive value (NPV) of preoperative aspiration culture were calculated. Then, the discordance between preoperative aspiration culture and intraoperative SF culture was analyzed.

**Results:**

The sensitivity of preoperative aspiration culture was 81.29% compared to intraoperative synovial fluid cultures. Concordance was identified in 147 PJI (78.61%) patients and culture discordance occurred in 40 patients (21.39%). In these discordant PJI patients, 24 patients (60%) were polymicrobial and no intraoperative synovial fluid culture growth was found in 16 PJI cases (40%). Preoperative monomicrobial staphylococcus results had a sensitivity of and a specificity of 80.43% and 83.16%, respectively. Preoperative polymicrobial results had the lowest sensitivity.

**Conclusions:**

The intraoperative synovial fluid re-cultures are necessary if the preoperative aspiration culture is positive and the discordance between preoperative aspiration culture and intraoperative synovial fluid culture should be noted especially when *Streptococcus* spp. and more than one pathogen was revealed by preoperative aspiration culture.

Level of evidence: Level III.

## Introduction

Periprosthetic joint infection (PJI), is a serious complication after total joint arthroplasties (TJA) and lays a huge burden on patients, surgeons, and healthcare systems worldwide [1–3]. Unfortunately, the incidence of PJI is increasing but the diagnosis and treatment of PJI remain challenging and controversial [3].

Identifying the offending pathogens in PJI patients is critical in initiating early antibiotic administration, choosing optimal surgical management strategy, and predicting prognosis [1, 2]. Preoperative joint aspiration is pivotal in the management of PJI patients because the preoperative identification of PJI pathogens and subsequent antibiotics sensitivity test (AST) can guide perioperative antibiotics administration and the selection of optimal surgery method [4].

However, to our knowledge, some studies revealed the difference between preoperative aspiration cultures and intraoperative tissue cultures but no studies comprehensively evaluated the concordance between preoperative aspiration culture and intraoperative synovial fluid culture [5]. Based on the EBJIS criteria of periprosthetic joint infection, both synovial fluid and at least five reliable tissue samples must be obtained using separate instruments and immediately transferred to the laboratory for culture. If a microorganism of high virulence was revealed in a single specimen from the patients, the PJI was diagnosed [6]. If a microorganism of low virulence was detected in a single specimen, PJI was highly suspected. However, according to these criteria, a problem was raised: are intraoperative synovial fluid re-cultures necessary if preoperative aspiration culture is positive. If a microorganism of high-virulence was detected in the preoperative aspiration culture, there may be no need to perform intra-op synovial fluid culture again because the PJI pathogen has been identified and extra tests increase the cost of PJI management. If a microorganism of low-virulence was detected in the preoperative aspiration culture, it may be necessary to perform an intra-op synovial fluid culture to further identify the specific PJI pathogen [7]. To address this problem, a retrospective study was conducted in a tertiary joint center to determine (1) the discordance between preoperative synovial fluid culture and intraoperative synovial fluid culture. (2) compared to intraoperative synovial fluid cultures, the diagnostic sensitivity and specificity, positive predictive value (PPV), and negative predictive value (NPV) of preoperative aspiration culture. Based on these data, we try to answer this question: Are intraoperative synovial fluid cultures necessary if the preoperative aspiration culture is positive.

## Materials and methods

### Patients

Institutional Review Board approval was attained before the commencement of this study and then, this study was performed in a tertiary care orthopedic center. A longitudinally institutional PJI database was queried from 2015 to 2019 for all PJI patients who were managed with surgeries (including DAIR, one-stage revision, and two-staged arthroplasty) at a single tertiary care joint center.

All PJI patients within this database met the 2011 MSIS (Musculoskeletal Infection Society) criteria (Table 1) [8] and the inclusion criteria were as follows:

1. PJI patients diagnosed by the 2011 MSIS criteria.
2. PJI patients managed with revisions at this tertiary joint center.
3. Patients with preoperative aspiration culture results from preoperative intra-articular aspiration within 90 days from revisions.
4. Only the latest aspiration culture results were included in this study if repeated joint aspirations were performed before revisions.

**Table 1:** The MSIS criteria after removing synovial fluid tests:

Major Criteria: (PJI is identified when one of the following criteria exist)
1) The identical pathogen was cultured in at least 2 samples
2) The presence of sinus that was communicated with the prosthesis
Minor Criteria: (PJI is identified when three of the following 6 criteria exist)
(1) Elevated serum C-reactive protein (CRP) AND erythrocyte sedimentation rate (ESR)
(2) A single positive culture
(3) Purulence in the affected joint.
(4) Positive histology analysis of periprosthetic tissue. (≥ neutrophil cells per HP)
(5) Elevated synovial fluid WBC count
(6) Elevated synovial fluid PMN%

Patients were excluded if the only available preoperative aspiration culture was performed at the outside hospital.

**Fig. 1:**
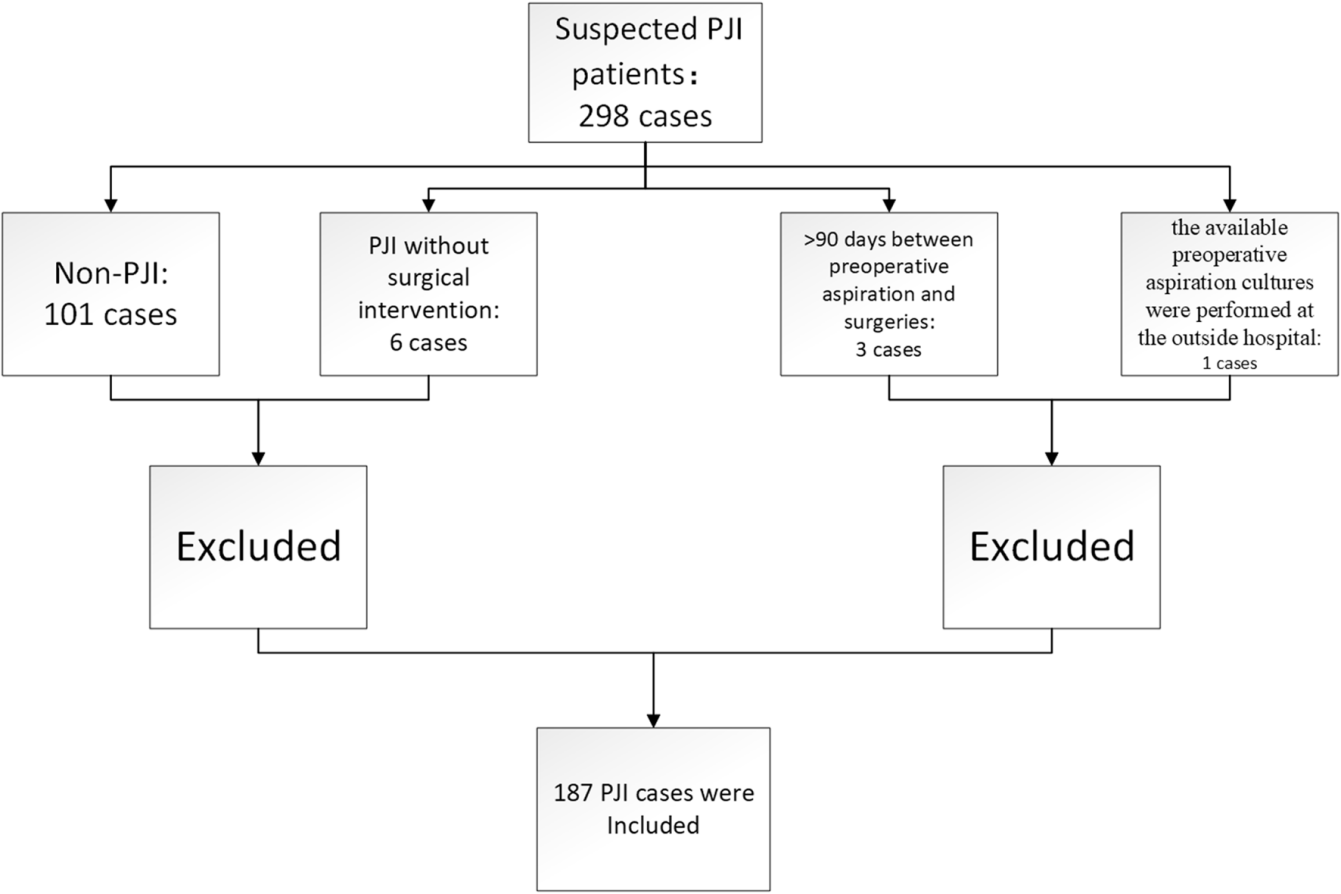
The flowchart of patients included in this study

The process of inclusion and exclusion was shown in Fig. 1.

### Chart review and data collection

Following demographic variables of included patients were also scrutinized and collected: the age, gender, BMI, joint, ASA scores, and comorbidities. Moreover, the culture results of preoperative and intraoperative specimens and corresponding AST were also recorded.

### Microbiological cultures

**Fig. 2:**
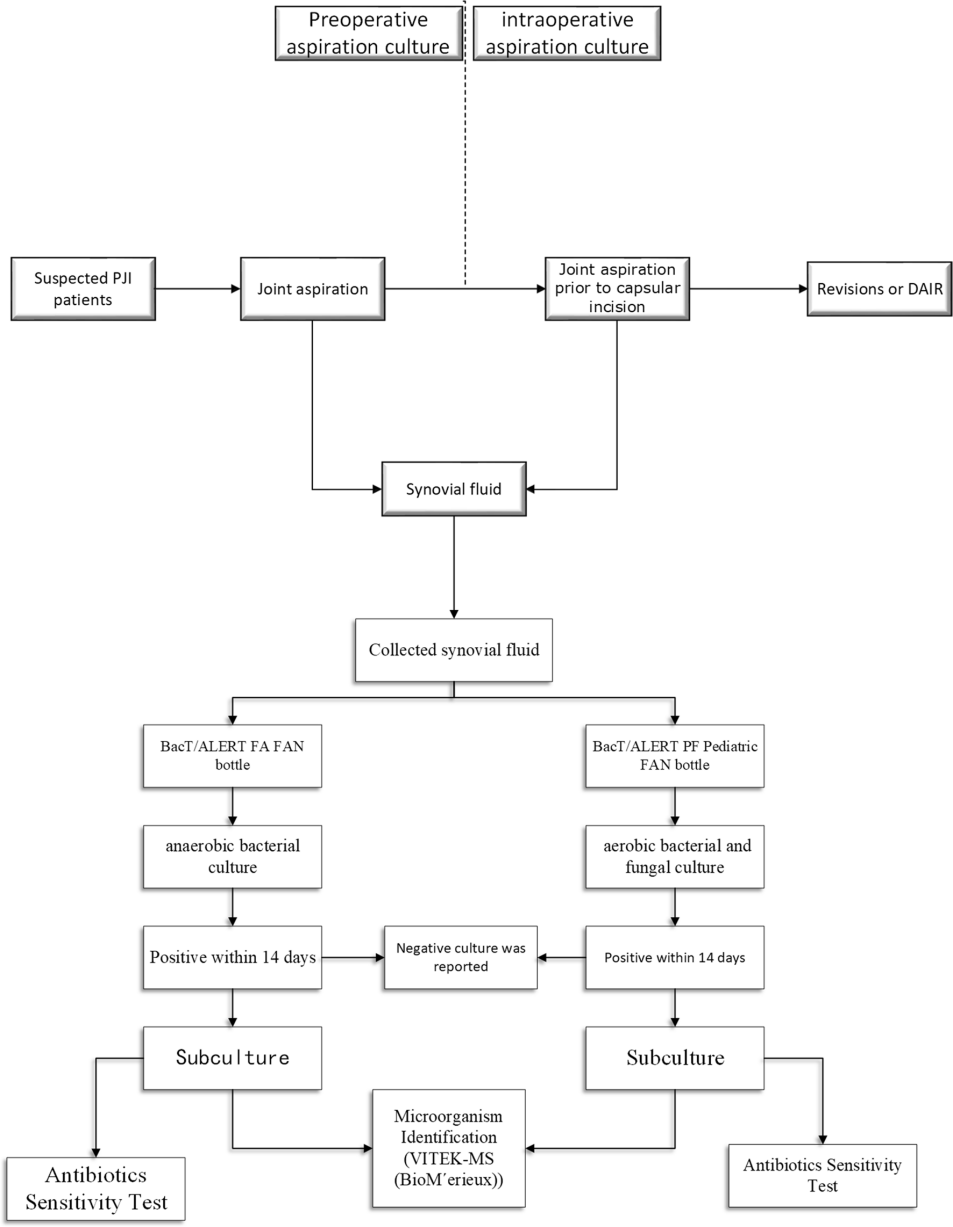
The design of this study

In this tertiary joint center, preoperative joint aspiration cultures were performed routinely in these PJI patients according to our institutional standards and this process had been described in previous studies [9]. All preoperative aspirations and the surgeries for PJI were done at the same treating institution, and the intraoperative aspiration before capsular incision was considered as the intraoperative aspiration for comparison to pre-op results. The process of preoperative aspiration culture and intraoperative synovial fluid culture were summarized in Fig. 2.

The obtained synovial fluid was injected into a BacT/ALERT FA FAN (fastidious antimicrobial neutralization) (bioMerieux) bottle for anaerobic bacterial culture and a BacT/ALERT PF Pediatric FAN (bioMerieux) bottle for aerobic bacterial and fungal culture. Each bottle was incubated for 2 weeks, and VITEK-MS (bioMerieux) was used for microorganism identification if pathogens were detected [10].

If a microorganism was revealed in either an aerobic bottle or an anaerobic bottle, this pathogen was recorded as the preoperative aspiration culture results. Then, antibiotic sensitivity tests were performed by disk diffusion according to the laboratory standard protocols.

### Antibiotics administration

According to the institutional protocols for PJI, if the patient received antibiotics within 2 weeks before joint aspiration, the joint aspiration was delayed until the patient was away from antibiotics for 2 weeks. Besides, no antibiotics were administrated before obtaining specimens sent for cultures in the PJI management surgeries.

### Definition

The definition of concordance and discordance was consistent with previous studies and the patients were divided into 2 categories: the concordant group, and the discordance group [4]. The concordant group was defined if both preoperative aspiration and intraoperative synovial fluid cultures revealed the same bacteria species with identical antibiotic resistance profiles. Otherwise, discordant cultures were defined.

To calculate the SE, SP, PPV, and NPV of preoperative aspiration culture and compare the difference between preoperative aspiration culture and intraoperative synovial fluid culture, the result of intraoperative synovial fluid culture was considered as the “gold standard ”.

If the discordant rate between preoperative aspiration culture results and the intraoperative synovial fluid cultures results were significantly (95%CI_lower_>0), the necessity of intraoperative synovial fluid cultures was defined.

### Statistical analysis

The baseline characterizes of the patients are described as continuous data and dichotomous data. The continuous data were presented as means or medians. The T-test is adapted to compare these data if the normal distribution is achieved for continuous variables. Otherwise, the rand-sum test is utilized. Dichotomous data were presented as frequencies and percentages. Then, these data were compared by chi-squared test or Fisher exact test. Statistical significance was defined if P < 0.05 and statistical analysis was performed on SPSS (IBM version: 22.0), Power BI (Microsoft version: 2019), and Excel (Microsoft version: 2018). Power analysis was performed on PASS 11.0.

## Results

### Demographic characteristics

Between 2015 and 2019, a total of 187 PJI patients managed with surgeries (including Debridement, Antibiotics and Implant Retention, one-stage revision, and two-staged arthroplasty) were included in this study finally. The PJI patients included in this study were divided into the concordant group and discordant group based on the concordance between preoperative aspiration cultures and intraoperative SF culture. The mean age in these two groups was 62.18 years and 61.5 years, respectively. The mean BMI in these two groups was 25.73 kg/m^2^ years and 25.11 kg/m^2^ years. The details on the demographic characteristics of these two groups were shown in Table 2.

**Table 2:** The demographic characteristics of PJI patients included in this study

	Total N = 187	Concordant group N = 147	Discordant group N = 40	P-value (concordant vs. discordant)
Age**	62.04 (60.09, 63.99)	62.18 (59.89, 64.48)	61.5 (57.86, 65.14)	0.434
Male**	98, 52.41%	77/147, 52.38%	21/40, 52.5%	0.989
BMI**	25.59 (25.08, 26.10)	25.73 (25.12, 26.30)	25.11 (23.96, 26.25)	0.446
Knee**	103, 55.08%	89/147, 60.54%	14/40, 35%	
Comorbidity				
Diabetes	19, 10.16%	17, 11.56%	2, 5%	0.374
IJD^a^	5, 2.67%	4, 2.72%	1, 2.5%	1.00
ASA score				
1	1, 0.53%	1, 0.68%	0%	1.00
2		128, 87.07%	39, 97.5%	0.081
3	19, 10.16%	18, 12.24%	1, 2.5%	0.081
4	0	0	0	1.00
Organisms				
Culture negative	46, 24.60%	37, 25.17%	9, 22.5%	0.728
*Staphylococcus* spp.	90, 48.13%	74, 50.34%	16, 4%	0.246
* Enterococcus* spp.	11, 5.88%	9, 6.12%	2, 5%	1.00
* Streptococcus* spp.	12,6.41%	4, 2.72%	8, 20%	0.001*
*Gram-positive* bacilli	4,2.14%	4, 2.7%	0	0.579
*Gram-negative* bacteria	11, 5.88%	10, 6.8%	1, 2.5%	0.462
Fungi	9, 4.81	8, 5.44%	1, 2.5%	0.687
Polymicrobial PJI	4, 2.13%	1, 0.68%	3, 7.5%	0.031

^a^IJD: Inflammatory Joint Diseases

**Values were given as means with the 95 %CI in the parentheses

*P < 0.05

### The concordance in PJI organism between preoperative aspiration and intraoperative synovial fluid culture

**Fig. 3:**
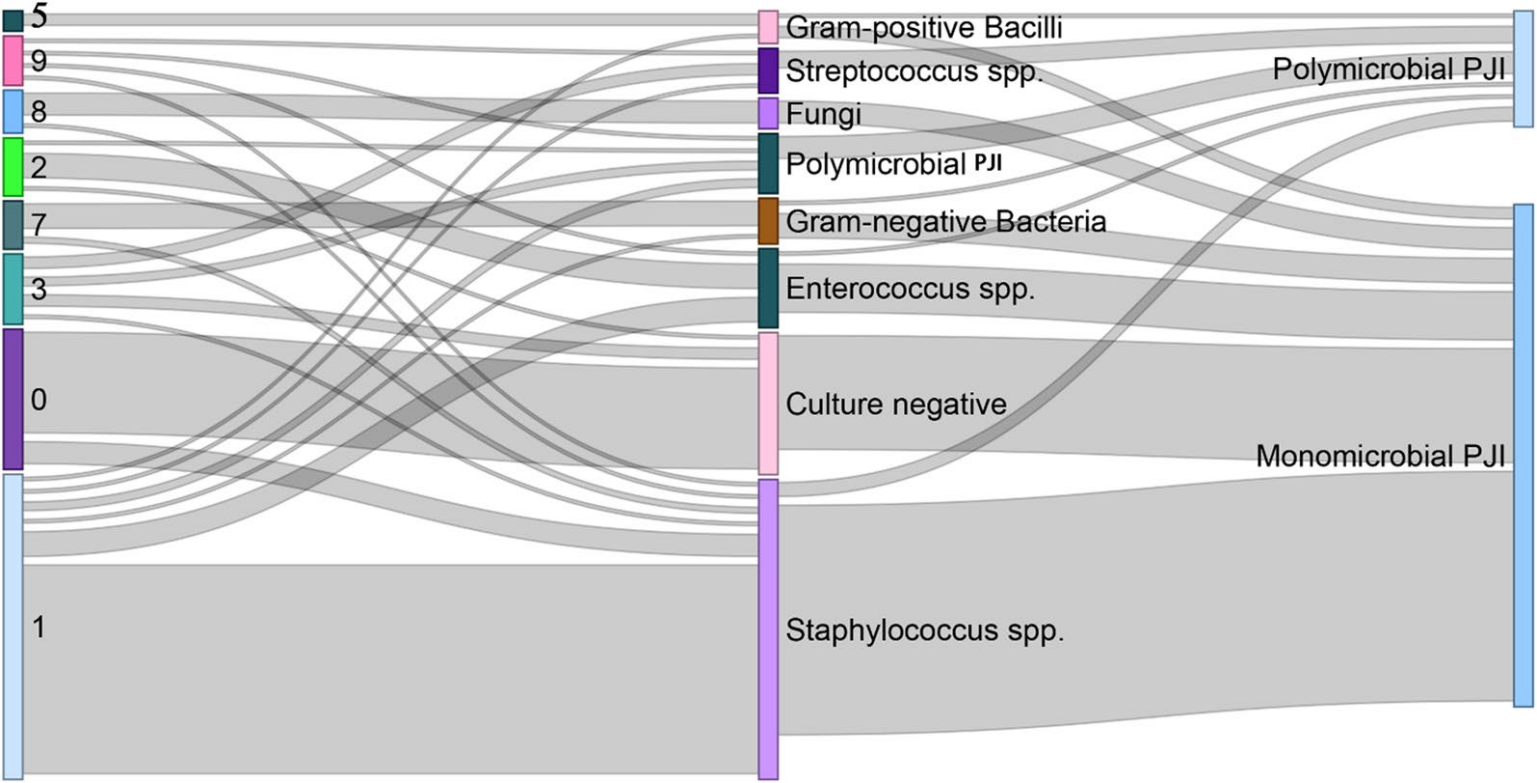
Sankey Diagram of Preoperative Aspiration and Intraoperative Synovial Fluid Culture Results. 1 = *Staphylococcus* spp.; 2 = *Enterococcus* spp.; 3 = *Streptococcus* spp.; 5 = Gram-positive Bacilli; 7 = Gram-negative Bacteria; 8 = Fungi; 9 = Polymicrobial PJI; 0 = Culture negative

The total concordant rate between preoperative aspiration cultures and intraoperative synovial fluid cultures was 85%. The concordant rate between these two cultures was about 82.22% in monomicrobial staphylococcus PJI patients. The concordant rate between these two cultures was 81.82% in monomicrobial enterococcus PJI patients. The concordant rate between these two cultures was 25% in polymicrobial PJI patients. The details about the concordant rate between these two groups were summarized in Table 3. Then, a Sankey plot was built to show the discordant pathogens between preoperative aspiration culture and intraoperative synovial fluid cultures in detail (Fig. 3).

**Table 3:** The Concordance and Discordance in PJI Organism between Preoperative Aspiration and Intraoperative synovial fluid Culture

Pathogens	Concordant rate, (n,%)	Discordant rate (%)	False negative rate of intraoperative synovial culture (n,%)
Culture-negative	37/46, 80.43%	19.57%	37/46, 80.43%
*Staphylococcus s*pp.	74/90, 82.22%	17.78%	9/90, 10%
*Enterococcus s*pp.	9/11, 81.82%	18.96%	1/11, 9.98%
*Streptococcus s*pp.	4/12, 33.33%	66.67%	4/12, 33.33%
Gram-positive Bacilli	4/4 100%	0	
Gram-negative Bacteria	10/11, 81.81%	18.18%	1/11, 18.18%
Fungi	8/9, 88.88%	11.12%	1/9, 11.12%
Polymicrobial PJI	1/4, 25%	75%	0

### The sensitivity, specificity, PPV, and NPV of positive preoperative synovial fluid culture by organism profile

The intraoperative synovial fluid cultures were considered as the “gold” standards of pathogens. Regarding the diagnostic performance of preoperative aspiration culture, monomicrobial staphylococcal PJI cases had a sensitivity of 80.43%, a specificity of 83.16%, a NPV of 81.44%, and a PPV of 82.22%. Streptococcus spp demonstrated a sensitivity of 66.67%, a specificity of 95.58%, a PPV of 33.33%, and a NPV of 98.86%. The enterococcus PJI cases had a sensitivity of 90%, a specificity of 98.87%, a PPV of 81.82%, and a NPV of 99.43%. The fungus PJI cases had a sensitivity of 100 %, a specificity of 99.44%, a PPV of 89%, and a NPV of 100%. The Gram-positive bacillus PJI cases had a sensitivity of 80%, a specificity of 100%, a PPV of 100%, and a NPV of 99.45%. However, polymicrobial PJI exhibited significantly lower sensitivity (50%). The details about the SE, SP, PPV, and NPV of preoperative aspiration culture and corresponding 95% confidence interval (CI) were summarized in Table 4.

**Table 4:** The sensitivity, specificity, PPV, and NPV of positive preoperative synovial fluid culture by organism profile

*Pathogens*	Sensitivity	Specificity	PPV	NPV
*Staphylococcus* spp.	80.43% (72.2 %, 88.7%)	83.16% (75.5%, 90.8%)	82.22% (74.2%, 90.3%)	81.44% (73.6%, 89.3%)
*Enterococcus* spp.	90% (67.4%, 100%)	98.87% (97.3%, 100%)	81.82% (54.6%, 100%)	99.43% (98.3%, 100%)
*Streptococcus* spp.	66.67% (12.5%, 100%)	95.58% (92.6%, 98.6%)	33.33% ( 2%, 64.6 %)	98.86% (97.3%, 100%)
Gram-positive Bacilli	80 % (24.5%, 100%)	100% (100%, 100%)	100% (100%, 100%)	99.45% (98.4%, 100%)
Fungi	100% (100%, 100%)	99.44% (98.3%, 100%)	88.89% (63.3%, 100%)	100% (100%, 100%)
Gram-negative Bacteria	100% (100%, 100%)	99.44% (98.3%, 100%)	90.91% (70.7%, 100%)	100% (100 , 100%)
Polymicrobial PJI	50% (5.3%, 94.7%)	100% (100%, 100%)	100% (100%,100%)	97.81% (95.7%, 100%)

The values were given as percentages with the 95 % CI (confidence interval) in the parentheses

## Discussion

The discordance between preoperative aspiration culture and intraoperative synovial fluid culture was about 20% (95% CI 18.9%–61.1%). Despite that the preoperative aspiration cultures were positive, it is still necessary to perform intraoperative synovial fluid re-cultures because this intraoperative culture can detect discordant pathogens in about 15% PJI patients compared to preoperative aspiration culture, especially when *Streptococcus* spp. (discordant rate: 33.33%) and more than one pathogen (discordant rate: 50%) was detected in preoperative aspiration cultures.

Pathogen identification is pivotal in the management of PJI since the information on PJI pathogens can guide perioperative antibiotics administration, treatment protocols and predict prognosis [3]. Although preoperative aspiration cultures are more convenient than intraoperative cultures, these culture results were obtained preoperatively, raising the possibility of suboptimal treatment protocol if discordant cultures were encountered [4, 5, 11]. Up to now, only one study investigated the concordance between preoperative aspiration and intraoperative SF culture, but these efforts were restricted to the limited number of PJI and septic arthritis patients [5]. We studied a large PJI cohort to comprehensively evaluate the difference between preoperative aspiration cultures and intraoperative synovial fluid cultures. A total of 187 patients were included in this study and power analysis was performed in this study. The study has 94% power, at an alpha of 0.05, to identify a difference of 15% in this PJI cohort. (The details were shown in the appendix). Therefore, the sample size of the study was enough to support our conclusion.

Inconsistent with previous studies, intraoperative cultures revealed more pathogens than preoperative aspiration cultures [4]. This fact can be attributed to multiple specimens sent for culture during revisions by which the sensitivity of cultures was improved and more pathogens were identified. It highlights the need for doctors to re-evaluate PJI pathogens during revisions and trim the antibiotic protocol after revisions.

The sensitivity of preoperative synovial fluid culture and intraoperative synovial fluid culture for PJI diagnosis was comparable. And the concordant rate between preoperative aspiration culture and intraoperative synovial fluid culture is about 80%. This result suggested that intraoperative synovial fluid culture was valuable despite preoperative SF culture was positive. If the preoperative synovial fluid culture results are negative, the intraoperative SF culture can identify pathogens in about 20% of preoperative culture-negative PJI patients. When the preoperative synovial fluid culture results were positive, the intraoperative SF culture can detect discordant pathogens and improve the detection rate of polymicrobial PJI in about 22% of preoperative culture-positive PJI patients.

In this study, some interesting findings were revealed. When *Streptococcus* spp. and Gram-positive bacilli were identified in preoperative aspiration cultures, the intraoperative SF culture was more likely to identified discordant pathogens compared to preoperative aspiration cultures. This result was consistent with previous studies where these pathogens were more common in polymicrobial PJI compared to other pathogens such as CNS [12, 13]. It suggests that intraoperative SF culture was strongly recommended when these pathogens were identified by preoperative aspiration cultures. Besides, in these patients, intraoperative culture results should be followed up promptly in a bid to adjust the antibiotics timely. Several studies also revealed that some specific pathogens such as *Streptococcus* spp. and *Enterococcus* spp. were associated with a higher risk of polymicrobial PJI [12, 14]. Many studies suggested that Gram-positive bacteria were the mostly commonly isolated ones in clinical practice. However, Gram-negative bacteria were not uncommon in PJI (accounting for about 20%) such as *E.coli*, *Pseudomonas aeruginosa* and *Acinetobacter baumannii*. And these pathogens can indicate the presence of polymicrobial PJI and adverse outcomes [15, 16].When these situations are encountered, repeated cultures and AST are also required. Besides, dalbavancin is a recently approved glycopeptide antibiotic with a long half-life and efficacy, especially against streptococci and staphylococci. This ABX showed good antibacterial activity against Gram-positive clinical isolates causing bone and joint infection in vitro. Considering its long half-life and efficacy, its use in the management of PJI is promising [17, 18].

In most preoperative aspiration culture-positive PJI cases, the pathogens were identified in two blood culture bottles (aerobic and anaerobic blood culture bottles) therefore PJI was identified based on the major criteria of 2011 MSIS criteria in these cases (two positive cultures of the same organism). However, in 6 cases, the pathogens were only identified in one blood culture bottle. In the PJI cases caused by fungi, these fungi were only identified in the aerobic blood culture bottles preoperatively. But these fungi revealed in aerobic blood culture bottles were further identified in subsequent intraoperative cultures. These findings suggested the high accuracy of this blood culture system when these blood culture bottles were used to enrich pathogens in synovial fluids.

Considering the difference between preoperative aspiration culture and intraoperative synovial fluid culture, the antibiotics were not recommended after aspiration but before surgery because the antibiotics administration can impair culture results. And the antibiotics were recommended to be administrated after specimen collection during surgeries.

There are still some limitations in this study. Firstly, this study was performed in a single joint center retrospectively and selection bias was imperative. Therefore, further examination of a multi-center study is necessary. Second, these PJI pathogens were identified by mass spectrum in this study and no biochemical identification results were compared. Some pathogens were classified into G-positive bacilli and no further identification was performed. These ambiguous identifications also added some bias to this study. Finally, the difference in outcomes between those with concordance vs. discordance was not evaluated in this study and this field needs to be explored further.

## Conclusions

The monomicrobial results of preoperative aspiration cultures can guide clinicians in the selection of treatment strategies in most monomicrobial PJI cases. But the discordant rate between preoperative aspiration culture and intraoperative SF culture should be noted. Intraoperative SF culture can identify the pathogens which haven’t been revealed by preoperative aspiration culture in about 20% of PJI cases. Therefore, the intraoperative synovial fluid re-cultures are necessary whether the preoperative aspiration culture is positive or not. Furthermore, special caution about the antibiotic administration and microbiological diagnosis are need when *Streptococcus* spp. and more than one pathogen was revealed by preoperative aspiration culture.

## Data Availability

The datasets used and/or analyzed during the current study available from the corresponding author on reasonable request.

## Abbreviations

TJA: Total joint arthroplasty
PJI: Periprosthetic joint infection
MSIS: Musculoskeletal Infection Society criteria

## References

[1] Gajdács M (2019). Anaerobes and laboratory automation: like oil and water?. Anaerobe.

[2] Jeverica S, El Sayed F, Čamernik P, Kocjančič B, Sluga B, Rottman M (2020). Growth detection of Cutibacterium acnes from orthopaedic implant-associated infections in anaerobic bottles from BACTEC and BacT/ALERT blood culture systems and comparison with conventional culture media. Anaerobe.

[3] Kapadia BH, Berg RA, Daley JA, Fritz J, Bhave A, Mont MA (2016). Periprosthetic joint infection. Lancet (London, England).

[4] Boyle KK, Kapadia M, Chiu YF, Khilnani T, Miller AO, Henry MW (2021). Are intraoperative cultures necessary if the aspiration culture is positive? A concordance study in periprosthetic joint infection. J Arthroplasty..

[5] Imagama T, Nakashima D, Seki K, Seki T, Matsuki Y, Yamazaki K (2021). Comparison of bacterial culture results of preoperative synovial fluid and intraoperative specimens in patients with joint infection. J Infect Chemother.

[6] McNally M, Sousa R, Wouthuyzen-Bakker M, Chen AF, Soriano A, Vogely HC (2021). The EBJIS definition of periprosthetic joint infection. Bone Joint J.

[7] Gajdács M, Urbán E (2020). Relevance of anaerobic bacteremia in adult patients: a never-ending story?. Eur J Microbiol Immunol.

[8] Parvizi J, Zmistowski B, Berbari EF, Bauer TW, Springer BD, Della Valle CJ (2011). New definition for periprosthetic joint infection: from the Workgroup of the Musculoskeletal Infection Society. Clin Orthop Related Res.

[9] Li R, Li X, Ni M, Zheng QY, Zhang GQ, Chen JY (2021). Anatomic landmark-guided hip aspiration in the diagnosis of periprosthetic joint infection. Orthopedics.

[10] Li R, Lu Q, Chai W, Hao LB, Lu SB, Chen JY (2019). Saline solution lavage and reaspiration for culture with a blood culture system is a feasible method for diagnosing periprosthetic joint infection in patients with insufficient synovial fluid. J Bone Joint Surg Am Volume.

[11] Kavolus JJ, Cunningham DJ, Rao SR, Wellman SS, Seyler TM (2019). Polymicrobial infections in hip arthroplasty: lower treatment success rate, increased surgery, and longer hospitalization. J Arthroplasty.

[12] Tan TL, Kheir MM, Tan DD, Parvizi J (2016). Polymicrobial periprosthetic joint infections: outcome of treatment and identification of risk factors. J Bone Joint Surg Am Volume.

[13] Wimmer MD, Friedrich MJ, Randau TM, Ploeger MM, Schmolders J, Strauss AA (2016). Polymicrobial infections reduce the cure rate in prosthetic joint infections: outcome analysis with two-stage exchange and follow-up ≥ two years. Int Orthop.

[14] Marculescu CE, Cantey JR (2008). Polymicrobial prosthetic joint infections: risk factors and outcome. Clin Orthop Related Res.

[15] Donadu MG, Zanetti S, Nagy ÁL, Barrak I, Gajdács M (2021). Insights on carbapenem-resistant *Acinetobacter baumanni*i: phenotypic characterization of relevant isolates. Acta Biologica Szegediensis.

[16] Gajdács M, Kárpáti K, Stájer A, Zanetti S, Gavino M, Donadu MG (2021). Insights on carbapenem-resistant *Pseudomonas aeruginosa*: phenotypic characterization of relevant isolates. Acta Biologica Szegediensis..

[17] Fiore V, De Vito A, Aloisio A, Donadu MG, Usai D, Zanetti S (2021). Dalbavancin two dose regimen for the treatment of prosthetic joint infections: new possible options for difficult to treat infectious diseases. Infect Dis (London, England).

[18] Pfaller MA, Flamm RK, Castanheira M, Sader HS, Mendes RE (2018). Dalbavancin in-vitro activity obtained against Gram-positive clinical isolates causing bone and joint infections in US and European hospitals (2011–2016). Int J Antimicrob Agents.

